# Reliability Criteria for Thick Bonding Wire

**DOI:** 10.3390/ma11040618

**Published:** 2018-04-17

**Authors:** Turker Dagdelen, Eihab Abdel-Rahman, Mustafa Yavuz

**Affiliations:** 1 Mechanical and Mechatronics Engineering, University of Waterloo, 200 University Ave W, Waterloo, ON N2L 3G1, Canada; myavuz@uwaterloo.ca; 2 System Design Engineering, University of Waterloo, 200 University Ave W, Waterloo, ON N2L 3G1, Canada; eihab@uwaterloo.ca

**Keywords:** reliability, electronic packaging, power modules, wire bonding, heel stress

## Abstract

Bonding wire is one of the main interconnection techniques. Thick bonding wire is widely used in power modules and other high power applications. This study examined the case for extending the use of traditional thin wire reliability criteria, namely wire flexure and aspect ratio, to thick wires. Eleven aluminum (Al) and aluminum coated copper (CucorAl) wire samples with diameter 300 μm were tested experimentally. The wire response was measured using a novel non-contact method. High fidelity FEM models of the wire were developed and validated. We found that wire flexure is not correlated to its stress state or fatigue life. On the other hand, aspect ratio is a consistent criterion of thick wire fatigue life. Increasing the wire aspect ratio lowers its critical stress and increases its fatigue life. Moreover, we found that CucorAl wire has superior performance and longer fatigue life than Al wire.

## 1. Introduction

Higher density power modules [1,2] are being introduced in many fields, such as wind turbines [3], traction systems of electric rail vehicles [4] and hybrid electric vehicles [5]. Thick bonding wires, with diameters in the range of 100–500 μm, are the main chip interconnection method for power modules. On the other hand, thin boding wires with diameters less than 100 μm are the main chip interconnection method for MEMS (Microelectromechanical systems) sensors and actuators. In both cases, they are also an important barrier to their long-term reliability [6,7,8,9]. The current approach to overcome this challenge is to improve wire material and/or wire design and processing [6]. For example, aluminum coated copper wire (CucorAl) has recently been introduced as a replacement to conventional aluminum (Al) wire [2]. Alloying and doping elements have also been introduced to improve performance under high temperature [10].

There are many bonding wire failure mechanisms. The most common of them are fatigue failures, namely heel crack and lift-off [1,11,12]. Joule heating due to high current flows subject the wires to thermal loads. Harsh environmental conditions, elevated operating temperature and mechanical vibrations, further exacerbate the severity of those loads. Over long-term, stress cycling results in fatigue failure at the heel [1,13,14].

The complexity of the electro-thermo-mechanical interactions wires undergo and the variability of their geometry make long-term prediction of their behavior, and therefore their design, quite challenging [2,11,15]. As a result, bonding wire design has come to relay on experimental evaluation of wire fatigue life under standard operating conditions and investigations of the impact of individual parameter variations. Different methods have been developed towards that end. The leading method, power cycling (PC), subjects power modules to periodic current loads until failure to determine wire fatigue life [2,7,8]. However, the power cycling period is long due to the large thermal time-constant of the wires; achieving steady-state conditions during the heating-cooling cycle requires around 3 s [1]. Therefore, accelerated mechanical fatigue tests were introduced to decrease the required time for PC tests [13,16,17,18].

An alternative design method, assumes that wire reliability is counter-proportional to peak point displacement (flexure) and proceeds to design wire loops that minimize flexure in order to maximize reliability. While most researchers have resorted to FEM analysis of wire under thermal loads, some have started to experimentally measure wire flexure under current loads using digital image correlation [19] and laser Doppler vibrometer (LDV) [20,21].

A common conclusion in literature posits that higher loops are more reliable and that they guarantee a longer fatigue life [2,13,15,16,22]. While PC tests have found that thin wires with an aspect ratio (height to length) ≥25% have less flexure and longer fatigue life [23], this assumption has not been critically examined for thick wires. In fact, Phillips and Harman [23] specifically note that their conclusion about thin wire reliability is not necessarily valid for thick wires.

The present paper aims to fill this gap by investigating the relationships among thick wire flexure, aspect ratio and fatigue failure. A FEM is first validated experimentally then used to locate and evaluate critical stresses along thick wires under various loading conditions. Specifically, we investigate the impact of wire material, loop geometry, and type of thermal load on wire flexure and critical stress.

## 2. Materials and Methods

### 2.1. Samples

**Figure 1:**
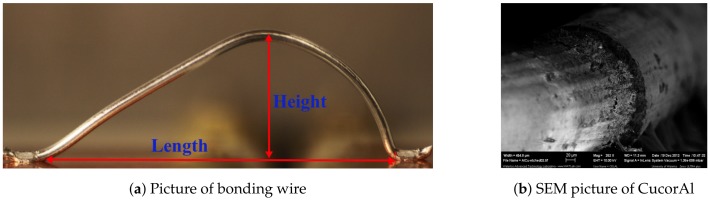
(**a**) Wire loop geometry and (**b**) layers of the CucorAl wire.

This study examines 300 μm diameter boding wire connecting two direct copper bonded pads (DCB) on a common alumina substrate [24]. The wire loop geometry is illustrated in Figure 1a . Loop height H is taken as the distance from the center of the wire at the peak point to the substrate and bond length L is taken as the foot to foot distance. Two wire materials were examined: aluminum (Al) [25] and aluminum-coated copper (CucorAl) [26]. The latter is made of a 25 μm thick Al coat on top of a copper core. Figure 1b shows a scanning electron microscope (SEM) image of a CucorAl wire where the Al coat has been etched out of the left half.

The loop height and bond length of the wire samples are listed in Table 1 (a). The mechanical and electrical properties of the wires are also listed in Table 1 (b). The alumina substrate and copper pad dimensions are listed in Table 2. The loop geometries adopted here are similar to those in standard use for power modules. Similar DCB substrates are also commonly used in power modules due to their high heat dissipation capabilities.

### 2.2. Experimental Methods

**Figure 2:**
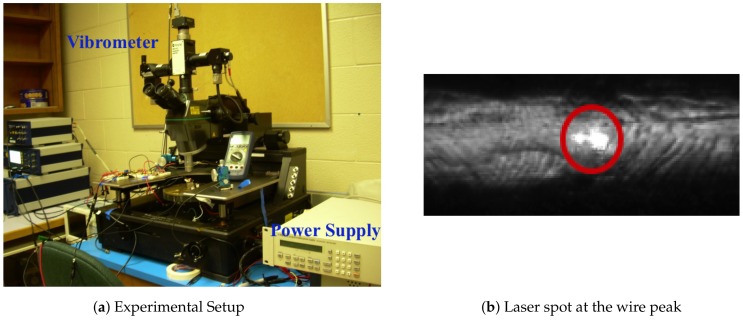
Measurement of wire displacement.

The experimental setup, Figure 2a, utilizes a Polytec MSV-400 laser Doppler vibrometer to obtain displacement of points along the wire span under thermal loading. This non-contact measurement technique evaluates the Doppler shift in the laser beam reflected from wire surface. A 10X objective lens is used to focus the laser beam on the wire surface. A picture of the laser spot is shown in Figure 2b. It is important to maintain the incident laser beam angle close to $90^{\circ }$ in order to maximize the power of the reflected laser beam and, thereby, the signal-to-noise ratio of the detection signal.

**Figure 3:**
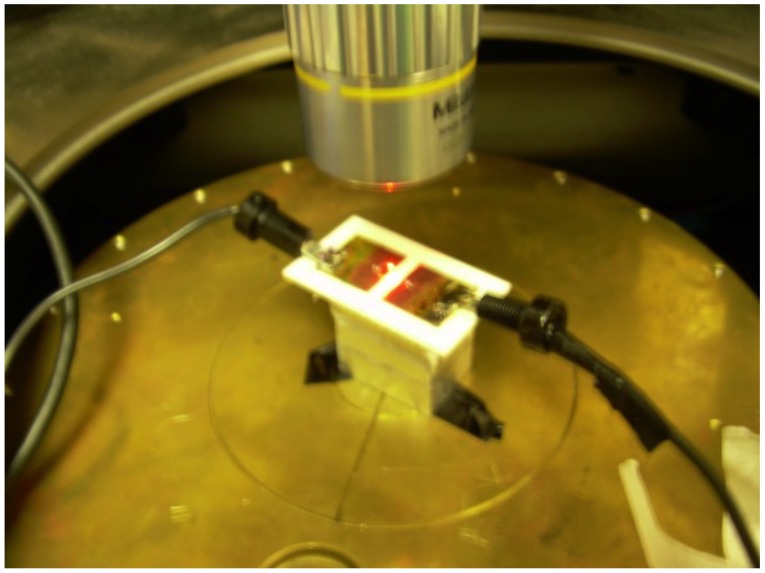
Wire sample under testing.

The probes of a DC power supply, Fluke PM2812 [27], were soldered to the pads on either side of the wire and used to pass a constant dc current through the wire. Figure 3 shows a picture of a wire sample and probes during testing. High current cables connected the power supply to the probes.

### 2.3. Numerical Methods

To guarantee high fidelity representation of the wire geometry, wire images were imported into SolidWorks and used to construct 3D models of the wires. For CucorAl wires, 3D models of the Cu core and Al coating were created separately then assembled. The wire models were then exported to Comsol for FEM simulation.

Electro-thermo-mechanical analysis was carried out on the wires. “Heat Transfer” module was used to determine their response under thermal loads caused by Joule heating. The multi-physics (electric current, heat transfer) modeling was achieved by defining terminals, grounds and the amount of current/heat values.

The substrate was not included in the FEM model. Instead, boundary conditions were imposed at the bottom surfaces of the wire feet representing the interface conditions. The mechanical boundary conditions held those surfaces fixed while the thermal boundary conditions set their temperature to room temperature, since the DBC substrates provide a large heat sink compared to the thermal mass of the wires. To account for natural convection, the heat transfer coefficient between the wires and air was set to 5 W/m${}^{2}$K. The electrical and thermal properties of the wires were defined as listed in Table 1 (b). Changes in wire properties with temperature were ignored.

## 3. Results and Discussion

### 3.1. FEM Model Validation

**Figure 4:**
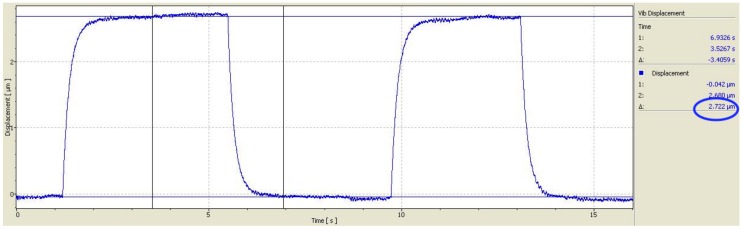
Experimentally measured wire flexure under a current pulse train with amplitude 6 A.

First, the current-flexure relationship was measured experimentally. Figure 4 shows the wire flexure for sample # 6 over two current pulses with an amplitude of 6 A. The flexure rises with each pulse and settles at a steady-state value of 2.72 μm.

**Figure 5:**
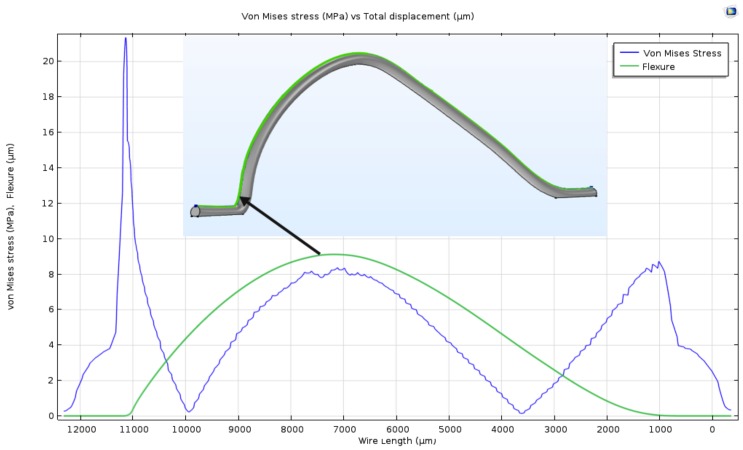
Vertical displacement and von Mises stress along the wire span under 10 A dc current.

Figure 5 shows the FEM prediction for the steady-state response of the same wire under a 10 A dc current. The vertical displacement of the wire along the green line in the inset of Figure 5 is plotted in green. Von Mises stress along the same line is also shown in blue. The predicted flexure was 9 μm whereas that measured experimentally was 8.6 μm, thereby validating the FEM model of the wire. The critical stress points along the wire were found at the inflection points in each of the wire feet where von Mises stress reached 21 MPa for foot 1 and 8.6 MPa for foot 2. The dominance of the stress at foot 1 is expected because of the higher curvature their compared to foot 2. This is consistent with the fact that most wire flexural fatigue failures occur at that foot [13].

### 3.2. Can Flexure Predict Wire Reliability?

**Figure 6:**
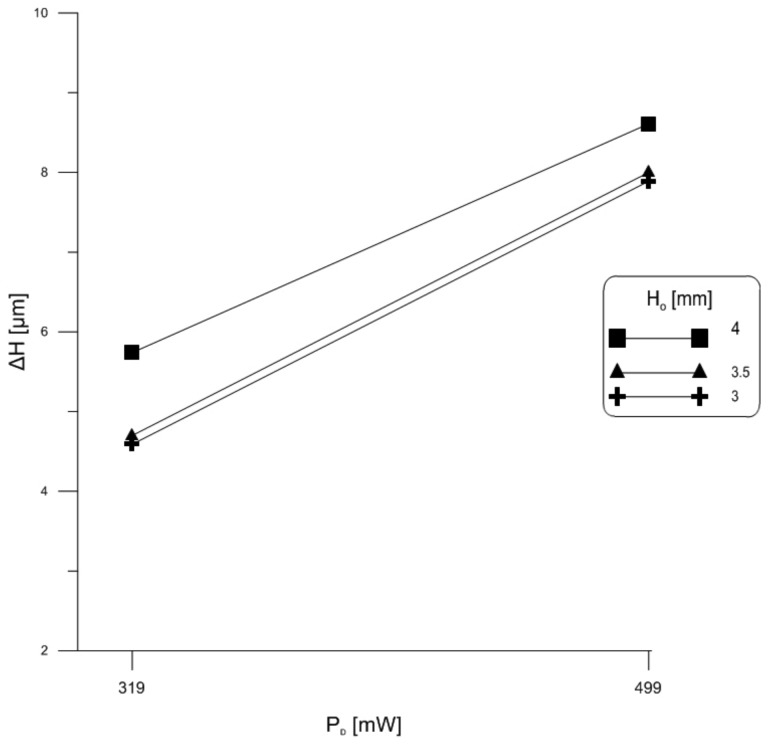
Wire flexure as a function of power.

First, we examined the relationship between wire flexure and power in samples # 2, 4 and 6. These samples are made of Al wire with the same length (L=10 mm) and heights of H=3 mm to 3.5 mm and 4 mm, respectively. FEM simulation was carried out on those wires when carrying a power of 399 mW and 499 mW. As expected, the predicted steady-state wire flexure, Figure 6, shows that wire deformation increases with the power it carries and, therefore, thermal load. On the other hand, we found that higher thick wires experience more flexure. This is counter to Phillips and Harman [23] findings for thin wires.

**Figure 7:**
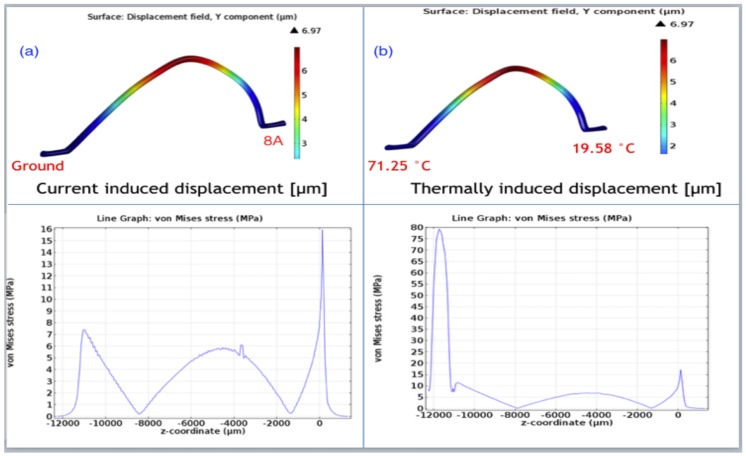
Vertical displacement and von Misses stress along the wire span under (**a**) dc current and (**b**) junction temperature difference.

The validated FEM model was also used to simulate the response of wire sample #7 to two different loading conditions: (i) a dc current of 8 A while both wire feet were held at room temperature and (ii) a junction temperature difference between the two feet in the absence of any current. In the second case, foot 1 temperature was held at 19.58 ${}^{\circ }$C and foot 2 at 71.25 ${}^{\circ }$C. Figure 7 shows that although the wire flexure was the same (6.97 μm) in both cases, the stress profiles along the wire span were quite different.

The critical point for the current carrying wire was at foot 1, Figure 7a, where von Mises stress was 16 MPa, while the critical point for the wire under junction temperature difference was at foot 2, Figure 7b, with von Mises stress of 17 MPa. This demonstration shows that wire flexure is a misleading indicator of the stress state, not only quantitatively but also qualitatively, since it fails to distinguish between wires where the critical point switches between foot 1 and foot 2.

### 3.3. Can Aspect Ratio Predict Reliability?

**Figure 8:**
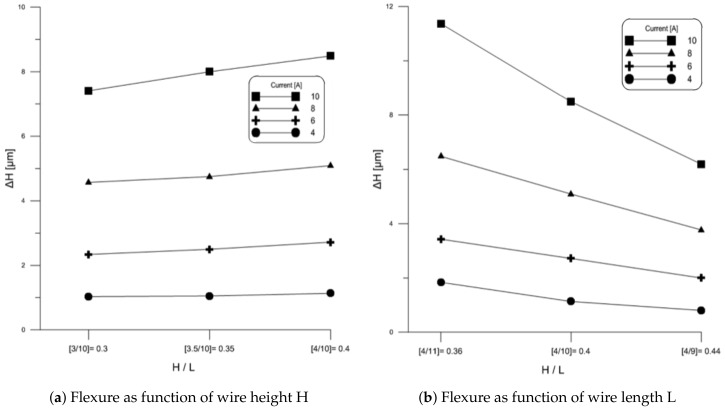
Impact of aspect ratio on Al wire flexure under dc current.

Another proposed measure of wire reliability is the aspect ratio H/L. In this section, we examine the impact of aspect ratio on Al wire flexure and stress. Figure 8a shows experimentally measured wire flexure when dc currents of 4 A, 6 A, 8 A, and 10 A was passed through wire samples # 2, 4 and 6. These samples have a common wire length of L=10 mm and heights of H=3,3.5 and 4 mm, respectively. The figure shows that increasing wire height and, therefore aspect ratio, increases wire flexure. Further, the increase in the slope of the wire height-flexure curve with current indicates that this relationship is nonlinear.

Figure 8b shows experimentally measured wire flexure when dc currents of 4 A, 6 A, 8 A, and 10 A was passed through wire samples # 5, 6 and 7. These samples have a common wire height of H=4 mm and wire lengths of L=9,10 and 11 mm, respectively. The figure shows that increasing wire length and, therefore decreasing aspect ratio, increases wire flexure. Further, the increase in the slope of the wire length-flexure curve with current indicates that this relationship is nonlinear.

Comparing the two sets of results, we find that aspect ratio can not be related to wire flexure. For a constant wire length *L*, increasing wire height *H* will increases flexure. Likewise, for a constant wire height *H*, increasing wire length will increase flexure.

**Figure 9:**
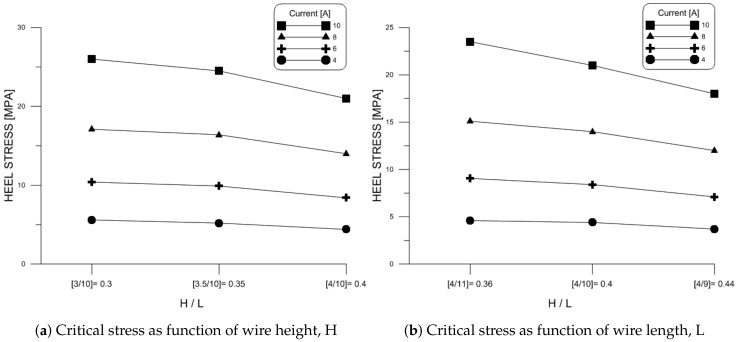
Impact of aspect ratio on the critical stress in Al wire under dc current.

Figure 9 shows the critical stress for the two sets of wire samples examined above predicted using FEM models of those wires under dc currents of 4 A, 6 A, 8 A, and 10 A. In all cases, it was found that the critical point occurred at the infliction point of foot 1.

Comparing the two sets of results show that aspect ratio is a consistent indicator of critical stress and, therefore, fatigue life and reliability under the same loading conditions. Increasing aspect ratio, by increasing wire height or decreasing wire length, lowers critical stress. On the other hand, flexure fails to predict reliability for variable wire geometries, even for the same type of loading conditions.

### 3.4. Al versus CucorAl Wires

**Figure 10:**
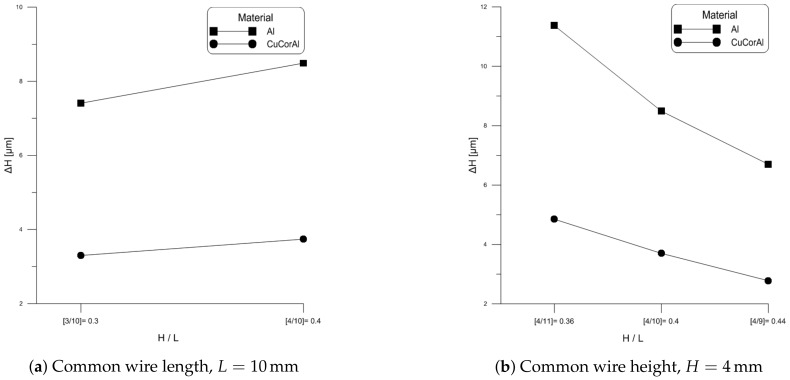
Comparison of wire flexure in Al and CucorAl wires with identical dimensions.

In this section, we compare the performance of Al and CucorAl wires. Figure 10a shows the experimentally measured steady-state wire flexure when a 10 A dc current was passed through wire samples # 2, 6, 8 and 10. All samples had the same length (L=10 mm). Samples # 2 and 8 share the same height (H=3 mm) but differ in material, Al and CuCorAl, respectively, likewise samples # 6 and 10 share the same height (H=4 mm) but differ in material, Al and CuCorAl, respectively. We found that CuCorAl undergoes lower flexure than Al wire which is consistent with its higher composite stiffness and higher electrical and thermal conductivities compared to AL wire.

In Figure 10b, we compare the experimentally measured steady-state wire flexure of samples # 5, 6, 7, 9, 10 and 11 under a dc current of 10 A . All of these samples had the same height of (H=4 mm), while their length varied at L=9,10 and 11 mm. Samples # 5, 6 and 7 were made of Al, while samples # 9, 10 and 11 were made of CuCorAl. Similar to the previous case, we found that CuCorAl wire undergoes lower flexure than Al wire.

**Figure 11:**
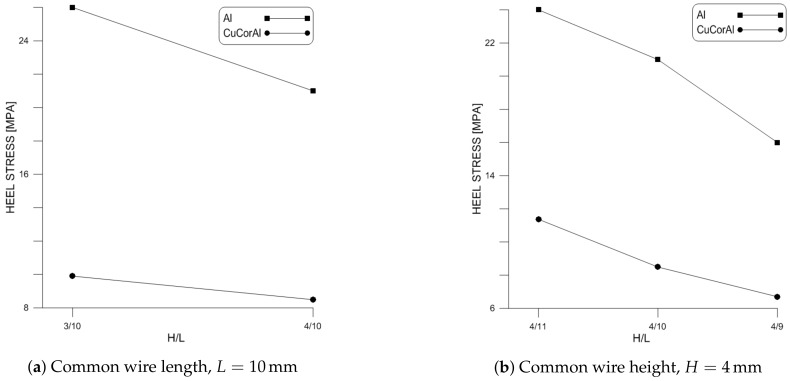
Comparison of critical stress in Al and CucorAl wires with identical dimensions.

Figure 11 shows the critical stress for the two sets of wire samples examined above obtained from FEM models of those wires under a dc current 10 A. Throughout all wire geometries, we found that CucorAl wire experienced lower critical stresses than Al wire with identical geometry due to its higher electrical and thermal conductivities.

## 4. Conclusions

In this study, an experimental technique was developed and used to study the performance of bonding wire under dc current. High-fidelity FEM models of bonding wire were also developed and validated experimentally. These tools were deployed to study the relationships among thick wire flexure, aspect ratio and fatigue life.

We found that thick wire flexure is not necessarily related to its aspect ratio and its stress state as far as the location and magnitude of critical stress. Therefore, it can not be used as an indicator of wire fatigue life. This is contrary to the case for thin wires, long established in literature, where a correlation exists among wire flexure, aspect ratio and fatigue life.

On the other hand, we found that wire aspect ratio is a consistent indicator of thick wire critical stress and fatigue life under the same loading conditions. Increasing aspect ratio lowers critical stress and increases fatigue life. Moreover, our comparison of Al and CucorAl wires shows that CucorAl wires exhibit lower critical stresses than Al wires under the same loading conditions and, therefore, should have superior performance and longer fatigue life.

## Figures and Tables

**Table 1:** Wire specimen.

**(a) Geometry**			
**Sample**	**Material**	**Height (mm)**	**Length (mm)**
1	Al	3	9
2	Al	3	10
3	Al	3	11
4	Al	3.5	10
5	Al	4	9
6	Al	4	10
7	Al	4	11
8	CucorAl	3	10
9	CucorAl	4	9
10	CucorAl	4	10
11	CucorAl	4	11
**(b) Properties**			
**Material**		**Al**	**CucorAl**
Diameter, d (μm)		300	300
Density, ρ (kg/m${}^{3}$)		2700	7082
Young’s modulus, E (GPa)		69	100
Resistivity, $p_{r}$ (μΩ·cm)		2.8	-
Thermal Cond., λ (W/mK)		230	-
Thermal Expansion Coeff., α (ppm)		28.3	-

**Table 2:** DCB dimensions.

Dimensions	Alumina (Al${}_{2}$O${}_{3}$)	Cu
Length (mm)	43	15
Width (mm)	25	15
Height (mm)	1	0.3
